# Systematic functional analysis of the Com pilus in *Streptococcus sanguinis*: a minimalistic type 4 filament dedicated to DNA uptake in monoderm bacteria

**DOI:** 10.1128/mbio.02667-23

**Published:** 2023-12-14

**Authors:** Jeremy Mom, Iman Chouikha, Odile Valette, Laetitia Pieulle, Vladimir Pelicic

**Affiliations:** 1 Laboratoire de Chimie Bactérienne, Aix-Marseille Université-CNRS (UMR 7283), Institut de Microbiologie de la Méditerranée, Marseille, France; GSK Vaccines, Siena, Italy

**Keywords:** type 4 pili, type 4 filaments, pilus assembly, genetic competence

## Abstract

Type 4 filaments (T4F) are a superfamily of versatile nanomachines, ubiquitous in prokaryotes, which use conserved multi-protein machineries to assemble and operate filamentous polymers of type 4 pilins. In the best-studied T4F, these machineries are complex, which has posed challenges to understanding the mechanisms of filament assembly and their multiple functions. Here, we report the systematic functional analysis of the Com pilus, a widespread T4F mediating DNA uptake during natural transformation in monoderm bacteria. Using *Streptococcus sanguinis*, we show that Com pili are *bona fide* type 4 pili (T4P), representing a new pilus sub-type. We show that with only eight components necessary for pilus assembly and functioning—all “core” proteins universally conserved in this superfamily—the Com pilus epitomizes a minimalistic T4F. We further demonstrate that core T4F components are sufficient for filament assembly. Paradoxically, akin to the more elaborate T4F, the Com pilus contains four minor pilins forming a complex, probably tip-located. Our results have global implications for T4F and make the Com pilus a model for elucidating fundamental processes underpinning filament assembly.

**IMPORTANCE:**

Type 4 filaments (T4F) are nanomachines ubiquitous in prokaryotes, centered on filamentous polymers of type 4 pilins. T4F are exceptionally versatile and widespread virulence factors in bacterial pathogens. The mechanisms of filament assembly and the many functions they facilitate remain poorly understood because of the complexity of T4F machineries. This hinders the development of anti-T4F drugs. The significance of our research lies in characterizing the simplest known T4F—the Com pilus that mediates DNA uptake in competent monoderm bacteria—and showing that four protein components universally conserved in T4F are sufficient for filament assembly. The Com pilus becomes a model for elucidating the mechanisms of T4F assembly.

## INTRODUCTION

Type 4 filaments (T4F) are a superfamily of nanomachines ubiquitous in bacteria and archaea, which mediate incredibly diverse functions, including adhesion, motility, protein secretion, and DNA uptake (1, 2). T4F use conserved multi-protein machineries to assemble and operate a filamentous polymer of type 4 pilins. How T4F are assembled and mediate such different functions remains poorly understood.

The best-characterized T4F are widespread virulence factors in diderm bacterial pathogens: type 4a pili (T4aP, where “a” denotes the sub-type) and type 2 secretion systems (T2SS). Although they produce very different filaments—long pili readily detectable on the cell surface (T4aP) or periplasmic endopili/pseudopili (T2SS)—these T4F use almost identical machineries (3). The ~15 conserved proteins form a multi-layered structure spanning the cell envelope (4). However, only four core components are universally conserved in T4F and are therefore likely to play a role in filament assembly (3): type 4 pilins (usually one major and several minor pilins), prepilin peptidase (PPase), extension ATPase, and platform protein. Pilins display a “lollipop” 3D architecture with a globular head impaled on an α-helix (α1) “stick” of ~50 residues (5). The protruding N-terminal half of this stick (α1N) corresponds to a stretch of hydrophobic residues, anchoring the pilins in the cytoplasmic membrane (CM) before polymerization. Filament assembly starts with the cleavage of the prepilin leader peptide by the PPase, a CM-embedded aspartic protease (6). Then, the CM-embedded platform and the cytoplasmic extension ATPase motor (7) extract pilins out of the membrane and polymerize them into filaments (3). The pilin α1-helices pack within the filament core, exposing the globular heads on the filament surface (8–13). Most of the other components of T4F machineries are involved after filament assembly (14).

An important function mediated by T4F in bacteria is DNA uptake, during which free DNA is captured from the environment and brought close to the CM (15). This is the first step in natural transformation, during which competent species take up DNA, translocate it across their CM, and incorporate it into their genomes. This is a property widespread in bacteria that promotes horizontal gene transfer (16). In monoderms, DNA uptake is mediated by a poorly characterized T4F, the Com pilus (17), which has been studied in *Bacillus subtilis* (18–24) and *Streptococcus pneumoniae* (25–29). This monophyletic T4F is widespread in Firmicutes (30). Although its major subunit ComGC displays a lollipop structure, it defines a novel pilin fold with an exclusively helical globular head (30). The Com pilus machinery is much simpler than T4aP and T2SS since it apparently consists of eight components: five pilins (ComGC, ComGD, ComGE, ComGF, and ComGG), PPase (ComC), extension ATPase (ComGA), and platform (ComGB) (24).

Although the simplicity of the Com pilus could be an asset for unraveling the mechanisms of T4F assembly, our poor understanding of this filament is an obstacle. There are many important outstanding questions about Com pili. (i) Are they *bona fide* pili as in *S. pneumoniae* (27, 31) or endopili as in *B. subtilis*, where filaments have not been visualized on the cell surface (32)? (ii) Are the above eight proteins required and sufficient for filament biogenesis? (iii) Are ComGD, ComGE, ComGF, and ComGG minor pilins? (iv) Do these minor pilins form a complex? Here, we have addressed these questions in *Streptococcus sanguinis*. This inhabitant of the human oral cavity, which frequently causes endocarditis (33), has recently emerged as a model monoderm species for T4F (3, 17). We finish by discussing the implications of our results for the T4F superfamily.

## RESULTS

### Com pili are *bona fide* T4P

*S. sanguinis* is a naturally competent species that has recently emerged as a model monoderm species for T4F (3, 17). It expresses T4aP, which mediate twitching motility (34) and adhesion to human cells (35, 36), but are dispensable for competence (34).

**FIG 1:**
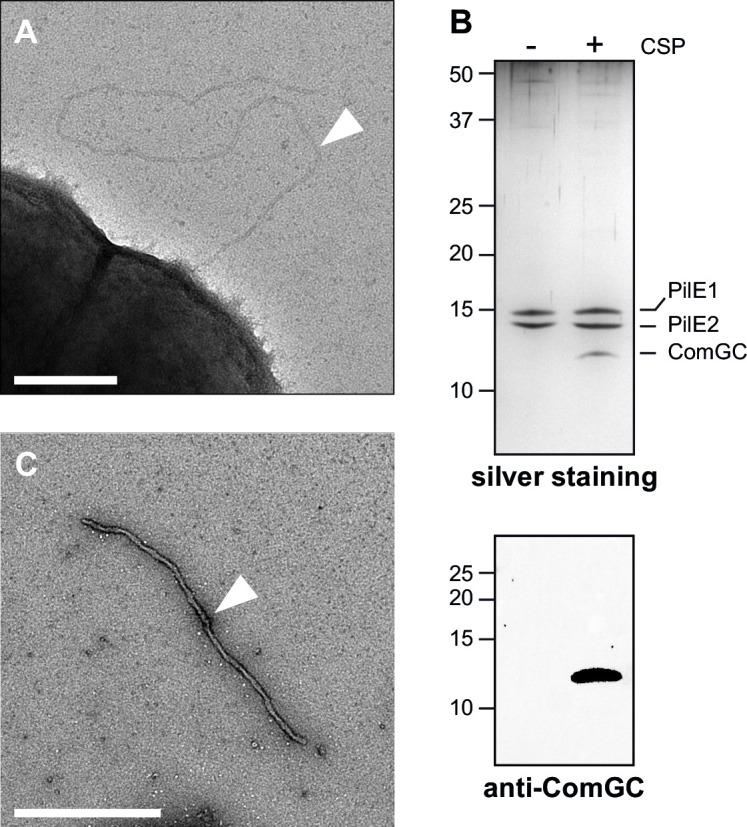
Visualization and purification of *S. sanguinis* Com pili. (**A**) Electron micrograph of a negatively stained Δ*pil* Δ*fim* mutant after CSP induction. The scale bar represents 200 nm. The white arrowhead indicates a Com pilus. (**B**) Silver staining (upper panel) and immunoblot analysis (lower panel) of purified Com pili. Samples were prepared ± CSP induction from cultures adjusted to the same OD_600_. Immunoblotting was performed using an anti-ComGC antibody. (**C**) Electron micrograph of negatively stained Com pili purified from the *Δpil* mutant. The scale bar represents 500 nm. The white arrowhead indicates a Com pilus.

DNA uptake in *S. sanguinis* is mediated by the Com pilus (34), which we started by characterizing morphologically to determine if they are *bona fide* pili. Since competence in *S. sanguinis* is under the control of a competence-stimulating peptide (CSP) (37), we imaged bacteria upon induction with synthetic CSP by transmission electron microscopy (TEM) after negative staining. To facilitate the identification of Com filaments, we used a Δ*pil* Δ*fim* mutant in which the two loci encoding T4aP (34) and sortase-assembled fimbriae (38) were deleted. This revealed long filaments exposed on the surface of *S. sanguinis* in the presence of CSP, with a classical T4P morphology (Fig. 1A). These flexible filaments are ~7 nm wide and ~1 µm long.

Next, we designed a robust pilus purification procedure to demonstrate that these filaments are Com pili. We adopted a procedure previously used for *S. sanguinis* T4aP (34). As assessed by silver staining after SDS-PAGE (Fig. 1B), three proteins between 10 and 15 kDa were predominant in pilus preparations made from the wild-type (WT) strain in the presence of CSP. The two larger proteins correspond to PilE1 and PilE2, the two major subunits of *S. sanguinis* T4aP that are produced constitutively (34). The protein around 10 kDa, which was not seen in the absence of CSP (Fig. 1B), corresponds to ComGC, as shown by immunoblotting (Fig. 1B). Pilus preparations from Δ*pil* in the presence of CSP contained an abundance of filaments, as shown by TEM, confirming that Com pili are *bona fide* pili. As illustrated in Fig. 1C, purified filaments displayed a morphology different from cell-associated pili, as they were much thicker (~ 15 nm) and straighter. This unexplained morphological change during purification was also observed for *S. sanguinis* T4aP (34, 39).

Together, these results show that *S. sanguinis* Com pili are *bona fide* pili composed primarily of ComGC subunits, with a typical T4P morphology.

### Competence is correlated to the transient production of Com pili

**FIG 2:**
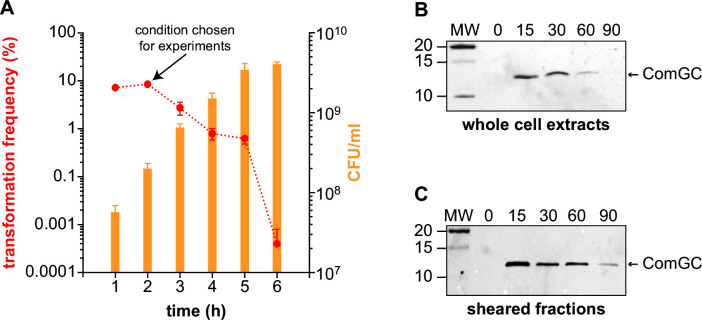
Correlation between competence, ComGC production and Com pilus production. (**A**) Competence development in relation to the growth phase. O/N cultures were adjusted to OD_600_ 1, diluted 100-fold in fresh THTH, and transformed after 1, 2, 3, 4, 5, or 6 h of growth at 37°C. Cell densities (CFU/mL) were determined by counting viable bacteria on non-selective plates (orange). Transformants were counted on plates containing streptomycin. Transformation frequencies (%) are the ratio of transformants relative to the number of viable bacteria (red). The results are the mean ± SD from three independent experiments. The condition chosen for further experiments—optimal transformation frequency at highest cell density—is indicated by an arrow. (**B**) Immunoblot detection of ComGC in cell extracts after 0, 15, 30, 60, and 90 min of induction with CSP. Extracts were quantified and equalized, and equivalent amounts of proteins were loaded in each lane. MW, molecular weight marker (in kDa). (**C**) Immunoblot detection of ComGC in sheared fractions after 0, 15, 30, 60, or 90 min of induction with CSP. Fractions were prepared from equal volumes of culture, and equal volumes were loaded in each lane.

We determined the competence parameters for strain 2908. We quantified competence over time using, as transforming DNA, a PCR product encompassing a mutant *rpsL* gene conferring resistance to streptomycin. As in other strains of *S. sanguinis* (40, 41), competence in 2908 was exquisitely dependent on cell density (Fig. 2A), maximal at early timepoints (reaching 8.52 ± 0.92% at 2 h), before decreasing dramatically.

We used immunoblotting to follow ComGC production in whole-cell extracts after induction with CSP (Fig. 2B). ComGC was detected 15 min after induction, and the signal peaked during the next 15 min before rapidly decreasing to become undetectable at 90 min (Fig. 2B). We similarly followed the production of filaments composed of ComGC over time (Fig. 2C). Filaments were sheared by vortexing and separated from the cells by centrifugation (42). After 15 min, ComGC was detected in sheared fractions, indicating that filament assembly occurs immediately upon production of the major pilin. ComGC levels remained high over the first 60 min, before decreasing significantly (Fig. 2C). Interestingly, at 90 min, while ComGC was undetectable in whole-cell extracts (Fig. 2B), it was detected in sheared fractions (Fig. 2C), suggesting that it is stabilized in filaments.

Together, these results show that there is a strong correlation in *S. sanguinis* between the transient production of Com pili and the development of competence, comparable to what has been reported in other species (16).

### Each *com* gene is required for Com pilus assembly and competence

**FIG 3:**
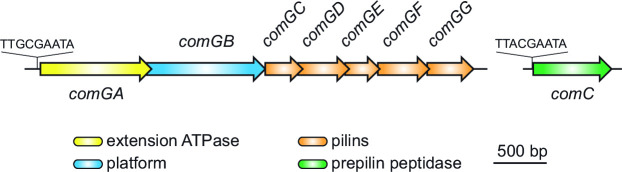
Genomic organization in *S. sanguinis* 2908 of the eight *com* genes involved in the synthesis of the Com pilus. The Com boxes preceding both transcription units—the binding site for the competence-specific σ factor ComX—are highlighted. The genes are drawn to scale. The corresponding proteins are listed at the bottom.

The eight *com* genes encoding Com pili in *B. subtilis* (22, 24) are present in the *S. sanguinis* genome, where they are organized in two transcription units (Fig. 3). Seven genes*—comGA*, *comGB*, *comGC*, *comGD*, *comGE*, *comGF*, and *comGG*—form an operon (19). The last gene, *comC*, is a distant loner (20). This genetic organization is widely conserved (Fig. S1). Both transcription units are preceded by a Com box (Fig. 3) (43)—TTnCGAATA—the binding site for the competence-specific σ factor ComX (44). The expression of *comX* is under the control of CSP (37). We characterized the role of the eight *com* genes in both DNA uptake and Com pilus assembly in *S. sanguinis*, which had never been done systematically before.

**FIG 4:**
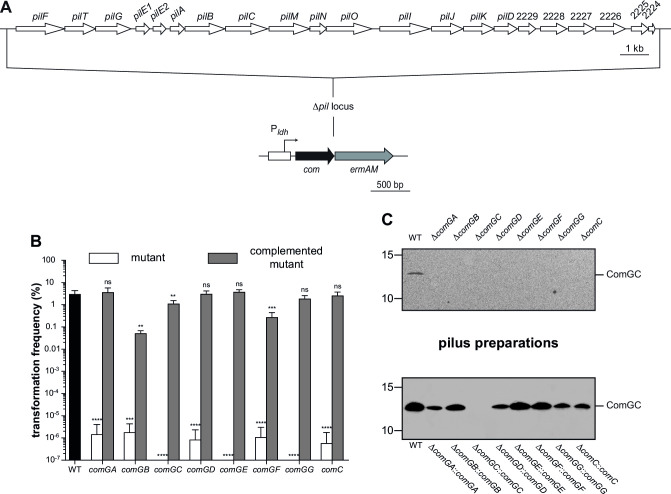
Systematic characterization of the role of the eight *com* genes in competence and Com pilus assembly. (**A**) Engineering a platform for complementation. First, we fused the complementing gene to *ermAM*, which confers resistance to erythromycin, and put them under the control of the constitutive lactate dehydrogenase promoter (P*_ldh_*) (46). This cassette was then spliced to the upstream and downstream regions of the *pil* locus, which encodes T4aP in *S. sanguinis* (34). The PCR product was transformed in 2908, and transformants were selected on plates containing erythromycin. As verified by sequencing, the *pil* locus was cleanly deleted and replaced by the cassette. (**B**) Competence of non-polar deletion mutants in the eight *com* genes and the corresponding complemented mutants. The WT strain is included as a control. Competence was quantified as in Fig. 2A. The results are the mean ± SD from at least three independent experiments (two experiments for *comGB*). We used Dunnett’s one-way ANOVA to compare the means to the control (WT): ns, not statistically different; **, *P* < 0.01; ***, *P* < 0.001; ****, *P* < 0.0001. (**C**) Com pilus production in non-polar deletion mutants in the eight *com* genes (upper panel) and in the corresponding complemented mutants (lower panel). Piliation was assessed by immunoblotting using an anti-ComGC antibody on pilus preparations made from equal volumes of culture. Equal volumes were loaded in each lane. The WT strain is included as a control.

Using a non-polar mutagenesis method (34, 45), we deleted each *com* gene in 2908. Considering that seven genes are expressed in a large operon (Fig. 3), it was crucial to complement the mutants to confirm that phenotypic alterations were not due to polar effects on downstream genes. This required the design of a novel complementing platform (Fig. 4A). In brief, complementing genes were placed under the transcriptional control of the highly expressed lactate dehydrogenase promoter (P*_ldh_*) (46) and inserted in the genome at the *pil* locus encoding T4aP (34), which was entirely deleted to facilitate the observation of Com pili (Fig. 4A). Since the Δ*com* mutants are non-transformable (see below), construction of complemented strains was done by first transforming the P*_ldh_ com-ermAM* cassette in the WT, before deleting the corresponding *com* gene.

Transformation was dramatically affected in each Δ*com* mutant (Fig. 4B). Whereas 3.10 ± 1.22% of WT cells were transformed, the mutants exhibited at least six orders of magnitude decreases in transformation (Fig. 4B). Importantly, competence was restored, most often at WT levels, when Δ*com* mutants were complemented (Fig. 4B), demonstrating that the non-transformable phenotypes are not due to polar effects.

We next determined whether the Δ*com* mutants are piliated by analyzing pilus preparations by immunoblotting using an anti-ComGC antibody. As seen in Fig. 4C, pilus production was abolished in each mutant, although ComGC was produced at WT levels, except obviously in Δ*comGC* (Fig. S2). When mutants were complemented piliation was restored, except for Δ*comGC::comGC* (Fig. 4C). Interestingly, ComGC was produced in Δ*comGC::comGC* at levels lower than in WT (Fig. S3). This shows that ComGC levels too low to lead to detectable pili can allow substantial DNA transformation.

Together, these results demonstrate that the eight *com* genes are required for both the production of Com pili and competence.

### The *com* genes are sufficient for Com pilus assembly

**FIG 5:**
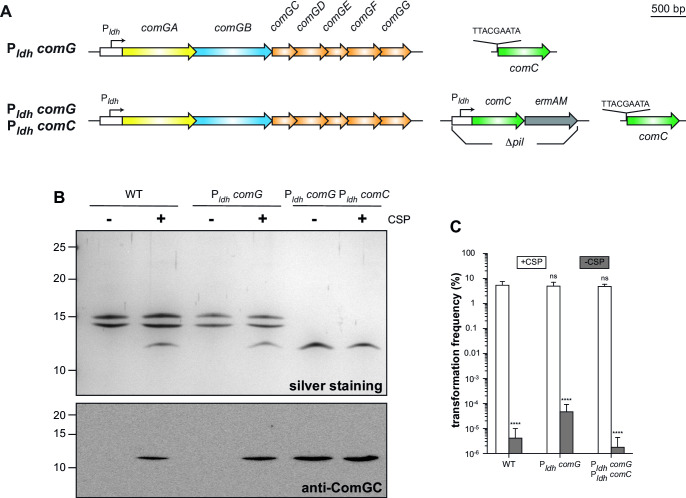
The eight *com* genes are sufficient for Com pilus assembly. (**A**) Engineering a strain expressing Com pili constitutively. First, we used the allelic exchange to replace the promoter of the *comG* operon by P*_ldh_*, generating the intermediate strain. We then inserted a P*_ldh_ comC-ermAM* cassette *in lieu* of the *pil* locus, generating the final strain. The final strain retains the native *comC* gene. (**B**) Silver staining (upper panel) and immunoblot analysis (lower panel) of purified Com pili from WT, intermediate/final strains. Samples were prepared ± CSP induction from cultures adjusted to the same OD_600_. Immunoblotting was performed using an anti-ComGC antibody. Molecular weight markers (in kDa) are indicated on the left. (**C**) Competence of WT and intermediate/final strains ± CSP. Competence was quantified as in Fig. 2A. The results are the mean ± SD from at least three independent experiments. We used Dunnett’s one-way ANOVA to compare the means to the control (WT in the presence of CSP): ns, not statistically different; ****, *P* < 0.0001.

Approximately 200 genes are under the control of CSP and ComX (43). It was thus possible that some of these other genes could be involved in the assembly of Com pili. We therefore engineered a strain expressing the *com* genes constitutively to determine whether they were sufficient for producing Com pili. We constructed this strain in two steps (Fig. 5A). First, we swapped the promoter of the *comG* operon with P*_ldh_*, generating the intermediate strain P*_ldh_ comG*. Then, in this strain, we inserted a P*_ldh_ comC-ermAM* cassette at the *pil* locus, generating the final strain P*_ldh_ comG* P*_ldh_ comC* (Fig. 5A).

We showed by immunoblotting that ComGC production becomes independent of CSP in the intermediate strain (Fig. S4). However, the pilin was not processed in the absence of CSP until ComC production became independent of CSP in the final strain (Fig. S4). Accordingly, in the intermediate strain P*_ldh_ comG*, Com pili are produced only in the presence of CSP (Fig. 5B), while they are produced constitutively in the final strain P*_ldh_ comG* P*_ldh_ comC* (Fig. 5B). Finally, we tested competence by measuring transformation (Fig. 5C). We found that the Com pili produced constitutively by the final strain are capable of mediating transformation to WT-levels (Fig. 5C), showing that they are perfectly functional. However, CSP was still needed for competence because many of the other genes involved in natural transformation, at steps occurring after DNA uptake, are also under the control of CSP and ComX (43).

Together, these results show that the eight *com* genes are not only necessary but also sufficient for Com pilus assembly.

### The Com pilus is a minimalistic T4F consisting only of core components

**FIG 6:**
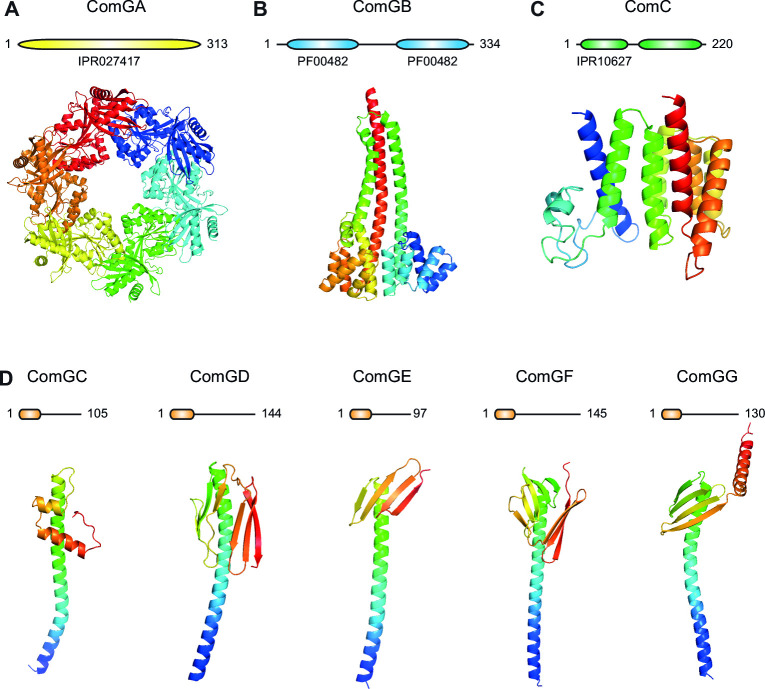
The Com machinery in *S. sanguinis* is a minimalistic T4F composed only of core proteins. Bioinformatic analysis of each protein: protein architecture drawn to scale (upper panel) and AlphaFold predicted structure in cartoon view (lower panel), rainbow-colored from blue (N-terminus) to red (C-terminus). (**A**) ComGA is the extension ATPase with an IPR027417 domain. The predicted structure is for the typical hexameric ring with a central pore. (**B**) ComGB is the platform protein with two PF00482 domains. (**C**) ComC is the PPase with two domains: an N-terminal methylase (IPR10627) and a C-terminal peptidase. (**D**) ComGC, ComGD, ComGE, ComGF, and ComGG are type 4 pilins, represented and modeled under their processed forms. They all start with a protruding α1N (orange rounded boxes) and display lollipop 3D architectures.

We previously noted that *S. sanguinis* Com proteins display sequence conservation with orthologs in *B. subtilis* and *S. pneumoniae* (30). We performed a thorough bioinformatic analysis by identifying protein domains using InterPro (47) and by predicting 3D structures using AlphaFold (48) (Fig. 6). All AlphaFold-generated models exhibit high confidence scores (Fig. S5).

The ComGA extension ATPase—the cytoplasmic motor powering T4F assembly (7)—exhibits a typical hexameric ring-shaped 3D structure with a central pore (Fig. 6A). The ComGB platform—the membrane-embedded T4F assembly hub (3) transmitting mechanical forces generated by the extension ATPase to membrane-embedded pilins—exhibits a typical “cherry” pair-like structure (3), where each cherry corresponds to a repeated domain (Fig. 6B). ComC the PPase—the aspartic protease that processes prepilins (6)—has a characteristic bi-modular structure with a C-terminal domain involved in the proteolysis of the prepilin leader peptides in prepilins and an N-terminal domain involved in the N-methylation of pilins (Fig. 6C) (49). The five type 4 pilins (ComGC, ComGD, ComGE, ComGF, and ComGG)—displaying characteristic N-terminal class 3 signal peptides (SP3) (Fig. S6) (5, 50)—exhibit typical lollipop architectures (Fig. 6D). The C-terminus of ComGG is the only portion with a low confidence score (Fig. S5). ComGC is the major pilin (32), while the remaining four are likely to be minor pilins. Interestingly, none of these has a purely helical globular head, which thus remains a distinctive feature of ComGC (30).

Together, these findings show that the Com machinery is composed only of core components universally conserved in T4F, making the Com pilus a minimalistic T4F significantly simpler than traditional models.

### ComGD, ComGE, ComGF, and ComGG are minor pilins

We focused on the five Com pilins, with the major pilin, ComGC, serving as a positive control. We tested whether ComGD, ComGE, ComGF, and ComGG are *bona fide* minor pilins, i.e., processed by the PPase ComC and incorporated in the pili as low abundance subunits.

**FIG 7:**
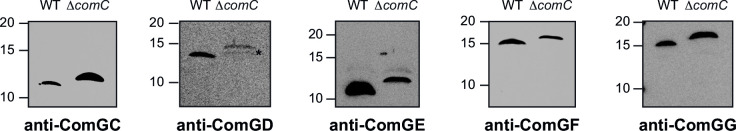
ComGD, ComGE, ComGF, and ComGG are genuine type 4 pilins processed by the PPase ComC. Immunoblot analysis of the processing by ComC of the leader peptide in the five Com pilins (ComGC is a positive control). We used specific antibodies, which were generated for this study. Whole-cell protein extracts were prepared from cultures started at the same OD_600_, and equivalent volumes were loaded in each lane (1/10 dilutions were used to detect ComGC). *Spurious band (or degradation product) detected using the anti-ComGD antibody. Molecular weight markers (in kDa) are indicated on the left.

After generating antisera against these four proteins, we detected them by immunoblotting in the WT strain and Δ*comC*. In the WT, processing by ComC is expected to generate mature proteins shorter than in Δ*comC*. As seen in Fig. 7, we indeed detected slightly longer proteins in *ΔcomC* than in the WT, which correspond to unprocessed prepilins, confirming that ComGD, ComGE, ComGF, and ComGG are all cleaved by ComC.

**FIG 8:**
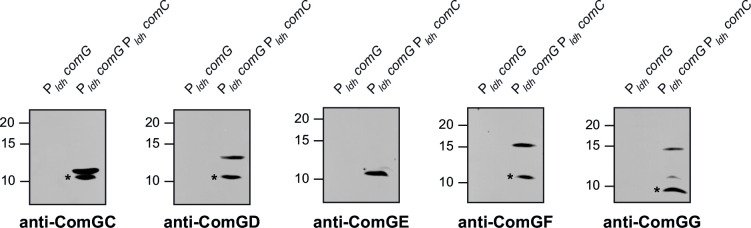
ComGD, ComGE, ComGF, and ComGG are minor pilus components. Immunoblot analysis of the co-purification of the five pilins (ComGC is a positive control) with Com pili. Pilus preparations from the final strain (expressing Com pili constitutively) and the intermediate strain (non-piliated) were made from cultures started at the same OD_600_, and equivalent volumes were loaded in each lane (1/10 dilutions were used to detect ComGC). *Spurious band around 10 kDa detected with several antibodies, corresponding to an unknown protein. Molecular weight markers (in kDa) are indicated on the left.

Unfortunately, our attempts to detect these four pilins in WT pilus preparations were repeatedly unsuccessful. This is not unexpected since WT pilus preparations are poorly concentrated because they are produced at very low bacterial density (16). In contrast, pilus preparations in P*_ldh_ comG* P*_ldh_ comC*, which are produced at higher cell densities, are more concentrated (Fig. 5B). We therefore used these latter preparations to repeat our immunoblots. As seen in Fig. 8, we could detect all four pilins, demonstrating that they are low-abundance components of the Com pili. As a control, we showed that these minor pilins were not detected in pilus preparations from the intermediate strain P*_ldh_ comG* (Fig. 8), which is non-piliated because ComC is not produced constitutively (Fig. 5B).

Together, these results show that Com pili are composed of five pilin subunits. ComGC is the major pilin, whereas ComGD, ComGE, ComGF, and ComGG are four minor pilins present in very low abundance.

### The four Com minor pilins form a complex likely to be tip-located

T4aP and T2SS both contain a set of four minor pilins—named with the letters H, I, J, K—essential for filament assembly (51, 52). These minor pilins interact to form a tip-located complex (53), which starts filament assembly since T4F are assembled from tip to base (52).

**FIG 9:**
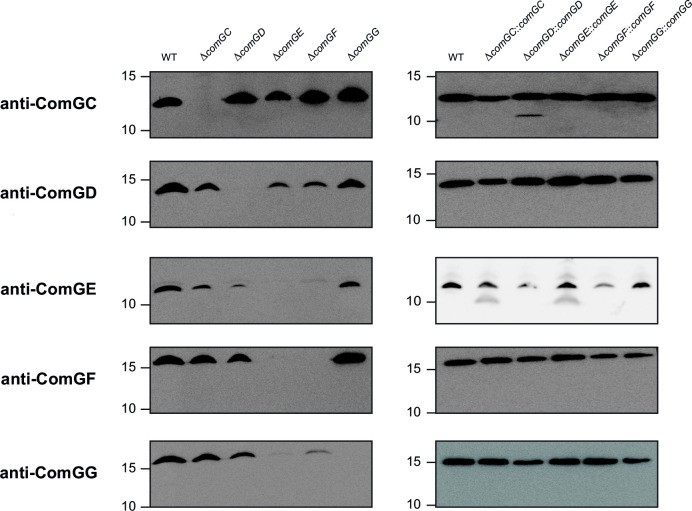
The four minor pilins interact to form a complex. Immunoblot analysis of the stability of each pilin in whole-cell protein extracts of pilin mutants (left panels) and mutants complemented with a WT copy of the deleted genes (right panels). The WT strain was included as a positive control. Extracts were quantified, and equivalent amounts of proteins were loaded in each lane. Molecular weight markers (in kDa) are indicated on the left.

We characterized the interactions between the four minor Com pilins. Since interacting proteins often exert mutually stabilizing effects (45), we used immunoblotting to assess the stability of every pilin in non-polar deletion mutants (Fig. 9). Critically, each protein (i) was detected in the WT, (ii) undetectable in mutants in which the corresponding gene was deleted, and (iii) detected again upon complementation. As seen in Fig. 9, several pilins displayed reduced levels in the absence of others, and their stability was restored upon the complementation of the interacting partners, ruling out the possibility of polar effects. In summary, ComGC is mainly unaffected by the absence of other pilins and has no major impact on their stability. ComGD stabilizes ComGE and is stabilized by ComGE and ComGF. ComGE and ComGF are strongly dependent on each other for stability, and the absence of either protein results in reduced levels of ComGG. Finally, ComGG has no detectable impact on the stability of other pilins.

**FIG 10:**
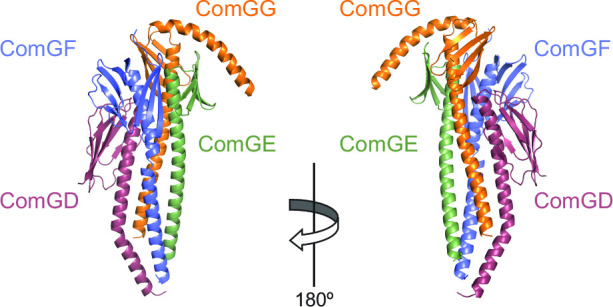
Structural model of the ComGD-ComGF-ComGE-ComGG complex of four minor pilins, predicted to be located at the tip of the Com pili. The AlphaFold-predicted structure is presented in 180° cartoon views, with the four subunits highlighted in different colors.

In other T4F, the H-J-I-K minor pilins form a tip-located complex (53), capped by the K subunit lacking a Glu_5_ (54, 55) (Fig. S7A). We modeled the complex between ComGD, ComGE, ComGF, and ComGG using AlphaFold (Fig. 10). This complex, which has a high confidence score (Fig. S5), is capped by ComGG, the only subunit lacking a Glu_5_. The next subunit is ComGE, followed by ComGF. The bottom subunit is ComGD, which links the complex with the filament. The GD-GF-GE-GG model is consistent with our interaction data (Fig. 9) and structurally similar to H-J-I-K in T2SS (55) (Fig. S7B).

Together, these results show that Com minor pilins interact to form a GD-GF-GE-GG complex, which is similar to H-J-I-K found in more elaborate T4F and thus likely to be at the tip of Com pili.

## DISCUSSION

T4F are a superfamily of nanomachines ubiquitous in bacteria and archaea (1, 2). Although T4F have been studied for more than 40 years—because they mediate important functions and are key virulence factors in numerous bacterial pathogens—the mechanisms of filament assembly and/or T4F-mediated functions remain poorly understood. One of the main reasons is that the best-characterized T4F are complex nanomachines with the most protein parts. Here, we took advantage of the inherent simplicity of T4F in monoderm bacteria (17) to systematically characterize one of the arguably simplest T4F: the Com pilus mediating DNA uptake. This led to several findings shedding light on important aspects of T4F biology.

Using *S. sanguinis*, which has emerged as a monoderm T4F model in the last decade (3, 17), we show that Com filaments are *bona fide* pili with a typical T4P morphology. This finding is important for several reasons. First, it is coherent with the notion that T4F act as molecular harpoons, capturing free DNA from the environment (56). Second, there was uncertainty about Com pili, as they have been described as pili in *S. pneumoniae* (27, 31) and endopili in *B. subtilis* (32). However, while this paper was under review, *bona fide* Com pili have been visualized in *B. subtilis* by fluorescence microscopy (57). Third, by confirming that Com pili are genuine T4P, we show that this monophyletic clade of T4F (2) corresponds to a new T4dP subtype (3). Critically, our finding that competence is possible at pilin levels too low for pili to be detected or visualized—which mirrors findings with *N. gonorrhoeae* T4aP that are involved in DNA uptake (58)—suggests that it will be possible for some Firmicutes to be competent in the absence of detectable Com pili.

The finding that Com pili are a minimalistic T4F composed only of core components has crucial implications for filament assembly. Our results provide strong experimental evidence that few pilins, one PPase, one extension ATPase, and one platform protein constitute the minimal machinery for T4F assembly (3). Until a system with fewer minor pilins is discovered—if such a system exists—T4dP will remain the simplest-characterized T4F (2). Our results are in favor of a generally applicable model for T4F assembly. Prepilins, translocated across the CM by the Sec machinery (59, 60), remain anchored in the CM via the hydrophobic portion of α1N. Their cytoplasmic leader peptides (61) are then cleaved by the CM-embedded PPase. Pilins are then extracted from the CM and assembled into filaments by the extension ATPase and platform, which act as a spindle motor (3).

Our third notable finding is that the Com pilus, despite its simplicity, displays a complex of four minor pilins at its tip, like more elaborate T4F. The ComGD-ComGF-ComGE-ComGG complex is homologous to PilH-PilJ-PilI-PilK in T4aP and GspH-GspJ-GspI-GspK in T2SS (54, 55). These sets of pilins share several features: (i) they are encoded by an operon, (ii) the last subunit lacks a Glu_5_ residue, (iii) and they form a complex in the order H-J-I-K (GD-GF-GE-GG) at the tip of T4F (53). The lack of Glu_5_ in the complex-capping K subunit is coherent with the fact that this residue usually neutralizes the positively charged N-terminus of the preceding subunit. The broad conservation of this complex supports a key role that is likely to be conserved as confirmed by the finding that the *gspHIJK* genes from T2SS can promote T4aP assembly (52). However, these minor pilins might also play specific roles in different systems, as suggested by (i) significant structural differences in the filament-capping subunits (e.g., GspK has a large extra module grafted on its globular head) and (ii) the finding that H-I-J-K interacts with the PilC/PilY1 adhesin in T4aP (53) or the secreted effectors in T2SS (62). By analogy, in Com pili, GD-GF-GE-GG might interact with DNA.

In conclusion, we have shown that Com pili represent a novel T4dP subtype corresponding to a minimalistic T4F with the fewest protein parts. Our findings have important implications for all T4F—especially concerning the mechanisms of filament assembly—and they pave the way for further investigations that could dramatically improve our understanding of a superfamily of nanomachines playing a key role in prokaryotic biology.

## MATERIALS AND METHODS

### Bacterial strains and growth conditions

*Escherichia coli* DH5α was used for cloning. It was grown in liquid or solid lysogeny broth (LB) medium (Difco), containing, when required, 100 µg/mL spectinomycin (Sigma). We used standard molecular biology techniques (63). If needed, PCR products were cloned into pCR8/GW/TOPO (Invitrogen), and/or purified using a QIAquick PCR purification kit (Qiagen).

Todd Hewitt (TH) broth (Difco) was used to grow *S. sanguinis* as described (34), with minor modifications. TH plates were incubated in anaerobic jars (Oxoid) under anaerobic conditions. Liquid cultures in THTH—TH containing Tween and HEPES [0.05% tween 80 (Merck) to limit bacterial clumping and 100 mM HEPES (Euromedex) to prevent acidification of the medium]—were grown statically under aerobic conditions. When required, kanamycin (500 µg/mL), erythromycin (5 µg/mL), or streptomycin (100 µg/mL) was used for selection. When needed, 15 mM *p*-Cl-Phe (Sigma) was used for counterselection.

All the *S. sanguinis* strains used in this study (Table S1) are derivatives of the 2908 throat isolate (34). Strains were constructed as follows, and all were verified by PCR and sequencing. Genomic DNA was prepared from liquid cultures using the XIT Genomic DNA from Gram-Positive Bacteria kit (G-Biosciences). PCR was done using high-fidelity DNA polymerase (Agilent). Primers are listed in Table S2. Marked non-polar deletion mutants were constructed using a previously described splicing PCR (sPCR) strategy involving no cloning (34, 45). Each target gene was replaced by a promoterless *aphA3* gene conferring resistance to kanamycin.

The unmarked Δ*pil* mutant was constructed by sPCR using a previously described two-step methodology with pheS* as a counter-selectable marker (64). The unmarked Δ*fim* mutant was constructed in one step by transforming *S. sanguinis*, in the absence of selective pressure (see below), with an sPCR product in which the regions upstream and downstream of the target genes were spliced.

Mutants were complemented by designing a new complementation platform. Originally, we spliced the promoterless *comX* to the *S. sanguinis* lactate dehydrogenase promoter (P*_ldh_*) and cloned in pCR8/GW/TOPO. This P*_ldh_ comX* cassette was then spliced to a promoterless *ermAM* gene conferring resistance to erythromycin and cloned in pCR8/GW/TOPO. Finally, the regions flanking the *pil* locus (34) were spliced into P*_ldh_ comX-ermAM* and transformed into *S. sanguinis*, replacing the *pil* locus in the process. Subsequently, this strain was used to construct all the other complemented strains by generating three PCR products for each gene with compF/compR, compF1/compR1, and compF2/compR2 (Table S2), which were spliced and transformed into *S. sanguinis*.

To engineer a strain expressing Com pili constitutively, we first generated the P*_ldh_ comG* intermediate strain, in which the native promoter of the *comG* operon was replaced by P*_ldh_*. We amplified the P*_ldh_ comGA* locus from the corresponding complemented strain, to which we spliced the region upstream of the endogenous promoter of the *comG* operon. This sPCR product was transformed into *S. sanguinis* in the absence of selective pressure. Then, we transformed this intermediate strain with the P*_ldh_ comC-ermAM* cassette from the corresponding complemented strain, generating the P*_ldh_ comG* P*_ldh_ comC* strain in which all the genes necessary for the biogenesis of the Com pilus are under the control of P*_ldh_*.

### Transformation assays

*S. sanguinis* was routinely transformed as described (34), with minor modifications. In brief, bacteria grown O/N in THTH were diluted to OD_600_ 0.01 in pre-warmed THTH and incubated at 37°C for 2 h. We then induced competence in this culture by adding synthetic CSP at a final concentration of 300 ng/mL and took a 330 µL aliquot to which we added 300 ng of transforming DNA. After incubation for 1 h at 37°C, bacteria were grown on plates with suitable antibiotics to select for transformants. We used a similar protocol for non-selective transformation, the major difference being that bacteria were diluted 10^−7^-fold in THTH after O/N growth and were bath-sonicated before plating using a Bioruptor (Diagenode) at medium amplitude for 45 s to break bacterial chains. If colonies were non-clonal, as verified by sequencing, bath-sonication was repeated.

For quantifying competence, a similar protocol was used. After O/N growth, bacteria were diluted in THTH. Induction was performed with 227 ng/mL CSP, and we used 100 ng of a purified PCR product encompassing the *rpsL* gene from a 2908 mutant spontaneously resistant to streptomycin (34). This mutant gene contains a single nucleotide polymorphism conferring resistance to streptomycin (34). After bath-sonication, we performed serial dilutions that were spread on plates with and without streptomycin. Frequencies were determined as the number of Str^R^ transformants/total CFU.

### Visualization and purification of Com pili

Surface-associated Com pili in *S. sanguinis* were visualized by TEM after negative staining. After O/N growth, bacteria were diluted in THTH to OD_600_ 0.01 and grown to OD_600_ 0.04–0.08, before pilus production was induced during 20 min with 300 ng/mL of CSP. Bacteria were adsorbed for 3 min on glow-discharged carbon-coated grids (EMS) and fixed for 5 min with 2% glutaraldehyde. The grids were sequentially floated on 10 drops of pilus buffer (20 mM Tris, pH 7.5, 50 mM NaCl) and stained for 2 min with 2% aqueous uranyl acetate. The stain solution was gently drained off the grids, which were air-dried before visualization using a Tecnai 200 KV electron microscope (Thermo Fisher Scientific). Images were acquired with a Oneview 16 Megapixel camera (Gatan).

Com pili were purified by adapting a protocol previously used for *S. sanguinis* T4aP (34). Liquid cultures grown O/N in THTH were used to re-inoculate pre-warmed THTH at OD_600_ 0.01 and grown statically for 2 h. CSP was then added at a final concentration of 300 ng/mL, and induction was performed for 30 min. Bacteria were pelleted by centrifugation for 15 min at 4,149 *g* at 4°C. Pili were sheared after re-suspending bacterial pellets in ice-cold pilus buffer, either by vortexing for 1 min at full speed or by repeated pipetting up and down. Bacteria were then pelleted by two rounds of centrifugation at 4°C for 10 min at 9,220 *g*. Finally, pili were pelleted by ultracentrifugation at 100,000 *g* for 1 h at 4°C. The pellets were resuspended in pilus buffer by pipetting up and down.

### Preparation of protein extracts, SDS-PAGE, antisera, and staining/immunoblotting

To prepare whole-cell protein extracts, liquid cultures grown O/N in THTH were used to re-inoculate pre-warmed THTH at OD_600_ 0.01 and grown statically for 2 h. When specified, CSP was then added at a final concentration of 300 ng/mL, and induction was performed for 30 min. Bacteria were pelleted by centrifugation at 4°C for 15 min at 4,149 *g* or 20 min at 9,220 *g*. Pellets were re-suspended in 1 mL of ice-cold pilus buffer and disrupted in a FastPrep-24 5G (MPBio) as previously described (34). When preparing cellular extracts, pili were sheared and removed by centrifugation before disruption.

SDS-PAGE was carried out in Tris-Tricine buffer (65). The Precision Plus Protein All Blue Prestained Protein Standards (Bio-Rad) was used as a molecular weight marker. Gels were stained with the Pierce Silver Stain Kit (Thermo Scientific) or transferred onto Amersham Hybond ECL nitrocellulose membrane (GE Healthcare) and analyzed by immunoblotting. Immunoblotting was done using standard molecular biology techniques (63). Skim milk powder was used for blocking. Antisera against ComG pilins were produced by immunizing rabbits with a mixture of two different synthetic peptides (Eurogentec). Primary antibodies were used at 1/2,500 (anti-ComGC) or 1/1,000 (all other antibodies) dilutions. The secondary anti-rabbit HRP-conjugated secondary antibody (GE Healthcare) was used at 1/10,000. Detection was performed using Amersham ECL Prime Western Blotting Detection Reagent (GE Healthcare) or SuperSignal West Atto Ultimate Sensitivity Chemiluminescent Substrate (Thermo Scientific).

### Bioinformatics

DNA Strider (66) was used for routine analysis of protein sequences. We identified protein domains using InterProScan (47). We used AlphaFold (48) for modeling protein 3D structures. We usually generated five models per prediction, with a final relaxation step. The models were ranked according to per-residue confidence metrics: pLDDT (monomers) or ipTM + pTM (multimers). We used PyMOL (Schrödinger) for generating the figures.

## SUPPLEMENTAL MATERIAL

Supplemental material mbio.02667-23-s0001.pdf Supplemental figures and tables.

ASM does not own the copyrights to Supplemental Material that may be linked to, or accessed through, an article. The authors have granted ASM a non-exclusive, world-wide license to publish the Supplemental Material files. Please contact the corresponding author directly for reuse.
