# Supplementary material for: Systematic functional analysis of the Com pilus in Streptococcus sanguinis: a minimalistic type 4 filament dedicated to DNA uptake in monoderm bacteria
Source: mBio. 2023 Dec 14;15(1):e02667-23. doi: 10.1128/mbio.02667-23 (PMC10790768; doi:10.1128/mbio.02667-23)
Supplement: Supplemental material — Supplemental figures and tables. [file mbio.02667-23-s0001.pdf]

Systematic genetic analysis of the Com pilus in *Streptococcus sanguinis*: a minimalistic type 4 filament dedicated to DNA uptake in monoderm bacteria

Jeremy Mom, Iman Chouikha, Odile Valette, Laetitia Pieulle, Vladimir Pelicic#

Laboratoire de Chimie Bactérienne, Aix-Marseille Université-CNRS (UMR 7283), Institut de Microbiologie de la Méditerranée, Marseille, France

#Address correspondence to Vladimir Pelicic, vladimir.pelicic@inserm.fr

Table S1. Bacterial strains used in this study.

Name	Description	Source
2908	naturally competent throat isolate	(1)
Str^{R} 2908	spontaneous mutant resistant to streptomycin (*rpsL*$_{A167G}$)	(1)
Δ*pil* Δ*fim*	Δ*pil* Δ*fim*	this study
P_{ldh} *comX*	Δ*pil*::P_{ldh} *comX-ermAM*	this study
Δ*comGA*	Δ*comGA*::*aphA3*	this study
Δ*comGA*::*comGA*	Δ*comGA*::*aphA3* Δ*pil*::P_{ldh} *comGA-ermAM*	this study
Δ*comGB*	Δ*comGB*::*aphA3*	this study
Δ*comGB*::*comGB*	Δ*comGB*::*aphA3* Δ*pil*::P_{ldh} *comGB-ermAM*	this study
Δ*comGC*	Δ*comGC*::*aphA3*	this study
Δ*comGC*::*comGC*	Δ*comGC*::*aphA3* Δ*pil*::P_{ldh} *comGC-ermAM*	this study
Δ*comGD*	Δ*comGD*::*aphA3*	this study
Δ*comGD*::*comGD*	Δ*comGD*::*aphA3* Δ*pil*::P_{ldh} *comGD-ermAM*	this study
Δ*comGE*	Δ*comGE*::*aphA3*	this study
Δ*comGE*::*comGE*	Δ*comGE*::*aphA3* Δ*pil*::P_{ldh} *comGE-ermAM*	this study
Δ*comGF*	Δ*comGF*::*aphA3*	this study
Δ*comGF*::*comGE*	Δ*comGF*::*aphA3* Δ*pil*::P_{ldh} *comGF-ermAM*	this study
Δ*comGG*	Δ*comGG*::*aphA3*	this study
Δ*comGG*::*comGG*	Δ*comGG*::*aphA3* Δ*pil*::P_{ldh} *comGG-ermAM*	this study
Δ*comC*	Δ*comC*::*aphA3*	this study
Δ*comC*::*comC*	Δ*comC*::*aphA3* Δ*pil*::P_{ldh} *comC-ermAM*	this study
P_{ldh} *comG*	P_{ldh} *comGA-GB-GC-GD-GE-GF-GG*	this study
P_{ldh} *comG* P_{ldh} *comC*	P_{ldh} *comGA-GB-GC-GD-GE-GF-GG* Δ*pil*::P_{ldh} *comC-ermAM*	this study

Δ, deleted gene(s).
::, inserted gene(s).
pil, 22 kb cluster of genes encoding T4aP (1).
fim, 5.4 kb cluster of genes encoding sortase-assembled fimbriae (2).
aphA3, cassette encoding an aminoglycoside phosphotransferase conferring resistance to kanamycin.
ermAM, cassette encoding an rRNA methyltransferase conferring resistance to erythromycin.
P_{ldh}, promoter of the gene encoding the lactate dehydrogenase in 2908.

Table S2. Primers used in this study.

Name	Sequence*
**Construction of marked deletion mutants**	
aphA3-F	ATGGCTAAAATGAGAATATCACC
aphA3-R	CTAAAACAATTCATCCAGTAAAA
ΔGA-F1	agccattgaagcagtaggga
ΔGA-R1	GGTGATATTCTCATTTTAGCCATacatcctcctcaccttactat
ΔGA-F2	TTTTACTGGATGAATTGTTTTAGGGGAGGAGGTGTCGTTGATT
ΔGA-R2	agagcattgccgcataaagt
ΔGB-F1	actgtgaggaagccaaggtt
ΔGB-R1	GGTGATATTCTCATTTTAGCCATcttaggcgtagacaatttttttc
ΔGB-F2	TTTTACTGGATGAATTGTTTTAGTGTTTTACTTTATGCGGCAATGC
ΔGB-R2	gtcctgtcctccgtctgaaa
ΔGC-F1	tcttcagcagcggttttcac
ΔGC-R1	GGTGATATTCTCATTTTAGCCATtataaatgaacctccatattctga
ΔGC-F2	TTTTACTGGATGAATTGTTTTAGAGCTCAGGAAATATCAGCCAGA
ΔGC-R2	aagtccctgccatcttgtct
ΔGD-F1	ttatcatgctgggattgcgc
ΔGD-R1	GGTGATATTCTCATTTTAGCCATcttaattggcaaccgtttgag
ΔGD-F2	TTTTACTGGATGAATTGTTTTAGTTTCAGACGGAGGACAGGA
ΔGD-R2	tgttgaaaagctgcaaacgc
ΔGE-F1	aggagatcggagcagacatg
ΔGE-R1	GGTGATATTCTCATTTTAGCCATtctagcttgaagctgtcgtc
ΔGE-F2	TTTTACTGGATGAATTGTTTTAGCACGAAGGGGAGGTAGTCAT
ΔGE-R2	agtccgacttaccagcttcg
ΔGF-F1	cagcggtggtcaaggtagta
ΔGF-R1	GGTGATATTCTCATTTTAGCCATtttaaggtttttgaacacggatg
ΔGF-F2	TTTTACTGGATGAATTGTTTTAGAGGTTTAGAGAGGGAGTTCGTC
ΔGF-R2	catcagcttcctctccgtga
ΔGG-F1	atttcaggccggcagatttc
ΔGG-R1	GGTGATATTCTCATTTTAGCCATctcaactttcttcttccacac
ΔGG-F2	TTTTACTGGATGAATTGTTTTAGAGAGAAAGCGATTTTGGACGA
ΔGG-R2	agtgtgctcatccggactag
ΔC-F1	cctccgcctcaaaataagc
ΔC-R1	GGTGATATTCTCATTTTAGCCATacttatttattcgtaattttaaacag
ΔC-F2	TTTTACTGGATGAATTGTTTTAGCTGTCTTTTGTTTGGTGTGG
ΔC-R2	cgttgaaagtagaggcgt
**Construction of unmarked deletion mutants**	
pheS-F	ATGACGAAAACGATTGAAGAAC
aph-R	CTAAAACAATTCATCCAGTAAAA
Δ*pil*-F1	gagaaagcgacaaggaggtg
Δ*pil*-R1	GTTCTTCAATCGTTTTCGTCATttgtttctcctgtctgtgatttt
Δ*pil*-F2	TTTTACTGGATGAATTGTTTTAGCTTTGAACTCAGACAGAAAGGGG
Δ*pil*-R2	tacacatgatccccagccag
Δ*pil*-R3	CCCCTTTCTGTCTGAGTTCAAAGTTGTTTCTCCTGTCTGTGATTTT
Δ*pil*-F3	AAAATCACAGACAGGAGAAACAACTTTGAACTCAGACAGAAAGGGG
Δ*fim*-F1	gccaagcacctgactagtag
Δ*fim*-R1	GCTCGGCTCTTTTCTTTTCCgtttggtcttcgaggcat
Δ*fim*-F2	atgcctcgaagaccaaacGGAAAAGAAAAGAGCCGAGC
Δ*fim*-R2	attccaccgcgtcatcaatg
**Complementation of deletion mutants**	
P_{ldh} *comX-F1*	agccacgataacactcatag
P_{ldh} *comX-R1*	ACCTTCTCATAAGTAGTTCTAAAATCCATttctaaacatctccttatttttttagg
P_{ldh} *comX-F2*	cctaaaaaaataaggagatgtttagaaATGGATTTTAGAACTACTTATGAGAAGGT
P_{ldh} *comX-R2*	TTATAAAATCGTATTGTCTTGAAAATC
P_{ldh} *comXerm-F1*	cccgagccacgataacactc
P_{ldh} *comXerm-R1*	GTTCATtgataatatctcctTTATAAAATCGTATTGTCTTGAAAATC
P_{ldh} *comXerm-F2*	TTATAAaggagatattatcaATGAACAAAAATATAAAATATTCTC
P_{ldh} *comXerm-R2*	TTATTTCCTCCCGTTAAATAATAG
comp-F1	tacacatgatccccagccag
compX-R1	CTAtgagtgttatcgtggctcCTTTGAACTCAGACAGAAAGG
compX-F2	AGCCACGATAACACTCATAG
compX-R2	TTATTTCCTCCCGTTAAATAATAG
compX-F3	CTATTATTTAACGGGAGGAAATAAttgtttctcctgtctgtgattt
comp-R3	gagaaagcgacaaggaggtg
comp-F1	tacacatgatccccagccag
compGA-R1	GCAATTTCTTGAACCATttctaaacatctccttatttttttagg
compGA-F2	ggagatgtttagaaATGGTTCAAGAAATTGCAAAAGAAATGATCAGG
compGA-R2	GTTCATtgataatatctcctTTAGGCGTAGACAATTTTTTCGG
compGA-F3	CCGAAAAAATTGTCTACGCCTAAaggagatattatcaATGAACA

comp-R3	gagaaagcgacaaggaggtg
comp-F1	tacacatgatccccagccag
compGB-R1	CCTTTGCTCAGCACttctaaacatctccttattttttttagg
compGB-F2	ggagatgtttagaaGTGCTGAGCAAAGGCAGACCGAAAAAATTGTC
compGB-R2	GTTCATtgataatatctcctTTATAAATGAACCTCC
compGB-F3	GGAGGTTCATTTATAAaggagatattatcaATGAACA
comp-R3	gagaaagcgacaaggaggtg
comp-F1	tacacatgatccccagccag
compGC-R1	GTTAAGTTTTTCATttctaaacatctccttattttttttagg
compGC-F2	ggagatgtttagaaATGAAAAAACTTAACACCTTAAAAGTTCAAGCATTCACCC
compGC-R2	GTTCATtgataatatctcctTTAATTGGCAACCGTTTGAGTT
compGC-F3	CTCAAACGGTTGCCAATTAAaggagatattatcaATGAACAA
comp-R3	gagaaagcgacaaggaggtg
comp-F1	tacacatgatccccagccag
compGD-R1	CCACTGTGTTTTCCATttctaaacatctccttattttttttagg
compGD-F2	ggagatgtttagaaATGGAAAACACAGTGGCGAAACTCAAACGG
compGD-R2	GTTCATtgataatatctcctCTAGCTTGAAGCTGTCGTCTTTTTAAACTTGCC
compGD-F3	CGACAGCTTCAAGCTAGaggagatattatcaATGAACA
comp-R3	gagaaagcgacaaggaggtg
comp-F1	tacacatgatccccagccag
compGE-R1	CGTCTTTTTAAACTTGCCATttctaaacatctccttattttttttagg
compGE-F2	ggagatgtttagaaATGGCAAGTTTAAAAAGACGACAGC
compGE-R2	GTTCATtgataatatctcctTTAAGGTTTTTGAACACGGATGACTACC
compGE-F3	GGTAGTCATCCGTGTTCAAAAACCTTAAaggagatattatcaATGAACA
comp-R3	gagaaagcgacaaggaggtg
comp-F1	tacacatgatccccagccag
compGF-R1	CCTTGACTTTAAGGTTTTTGAACACttctaaacatctccttattttttttagg
compGF-F2	ggagatgtttagaaGTGTTCAAAAACCTTAAAGTCAAGG
compGF-R2	GTTCATtgataatatctcctTCAACTTTCTTCTTCCACACG
compGF-F3	GGAAGAAGAAAGTTGAaggagatattatcaATGAACA
comp-R3	gagaaagcgacaaggaggtg
comp-F1	tacacatgatccccagccag
compGG-R1	CCTCAACTTTCTTCTTCCACACttctaaacatctccttattttttttagg
compGG-F2	ggagatgtttagaaGTGTGGAAGAAGAAAGTTGAGG
compGG-R2	GTTCATtgataatatctcctTTAGTCCGACTTACCAGCTTCGTCC
compGG-F3	GGACGAAGCTGGTAAGTCGGACTAAaggagatattatcaATGAACA
comp-R3	gagaaagcgacaaggaggtg
comp-F1	tacacatgatccccagccag
compC-R1	GTATAGATGAATCATttctaaacatctccttattttttttagg
compC-F2	ggagatgtttagaaATGATTCATCTATACTTTTTTCTCC
compC-R2	GTTCATtgataatatctcctTCAGTAAAAGAGTAGGG
compC-F3	CCCTACTCTTTTACTGAaggagatattatcaATGAACA
comp-R3	gagaaagcgacaaggaggtg
**Construction of a strain expressing Com pili constitutively**	
P_{ldh} *GA*-F1	CTCCGTCAAAGATTATCATGG
P_{ldh} *GA*-R1	gatgtaaacgtttttacaaatggTTAAAGAGGCTTAGCGGTTTTT
P_{ldh} *GA*-F2	CTAAAAACCGCTAAGCCTCTTTAAccatttgtaaaaacgtttacatc
P_{ldh} *GA*-R2	TTAGGCGTAGACAATTTTTTTCGG
**Generating a PCR product for quantifying competence**	
rpsL-F	ggcaggtgtagctgtctttg
r*psL*-R	ctcttgctccatccagtcca

*Genes are in upper case; flanking regions are in lower case. Regions of complementarity for splicing PCR are underlined.

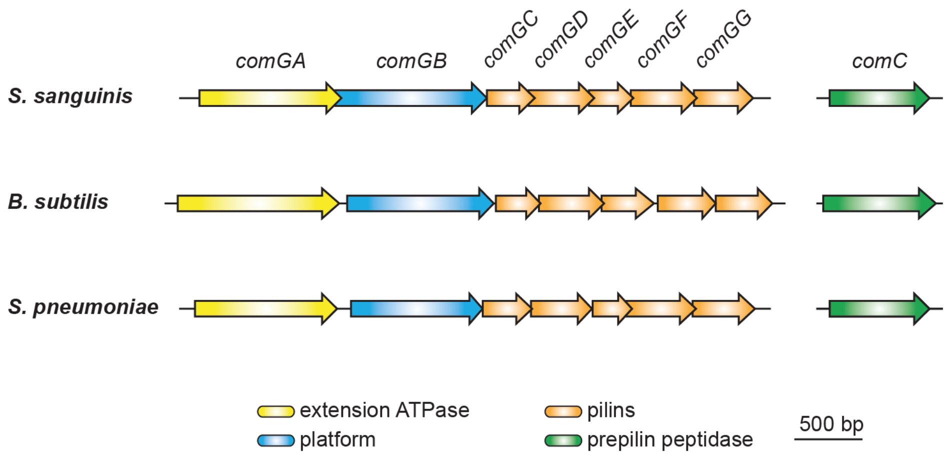

Fig. S1. Genomic organization of the genes involved in the synthesis of the Com pilus in model competent species. The genes, with the same color code as in Fig. 3, are drawn to scale. The corresponding proteins are listed at the bottom. The chosen strains are *S. sanguinis* 2908, *B. subtilis* 168 and *S. pneumoniae* TIGR4.

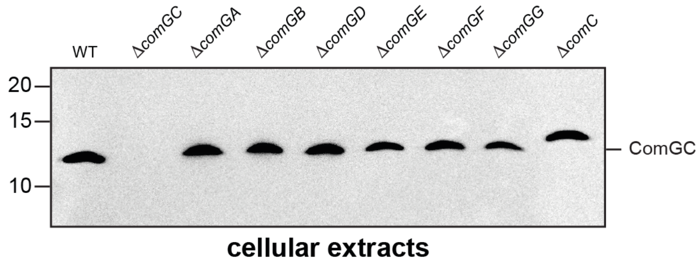

Fig. S2. ComGC production in deletion mutants in the eight *com* genes. Immunoblotting was performed on cellular extracts using an anti-ComGC antibody. The WT strain is included as a control. Extracts were quantified, equalized, and equivalent amounts of proteins were loaded in each lane. Molecular weight markers (in kDa) are indicated on the left.

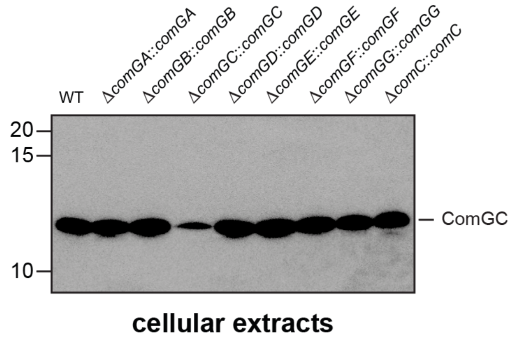

Fig. S3. ComGC production in deletion mutants in the eight *com* genes complemented with a WT copy of the corresponding genes. Immunoblotting was performed on cellular extracts using an anti-ComGC antibody. The WT strain is included as a control. Extracts were prepared from cultures equalized to the same OD_{600}, and equivalent volumes were loaded in each lane. Molecular weight markers (in kDa) are indicated on the left.

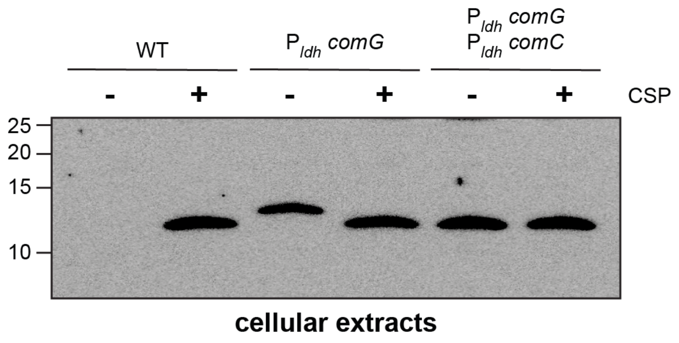

Fig. S4. Detection of ComGC by immunoblotting in a strain expressing Com pili constitutively. Cellular extracts were prepared +/- CSP induction from cultures of WT, P_{ldh} *comG* (intermediate strain), and P_{ldh} *comG* P_{ldh} *comC* (final strain) adjusted to the same OD_{600}. Immunoblotting was performed using an anti-ComGC antibody. Molecular weight markers (in kDa) are indicated on the left.

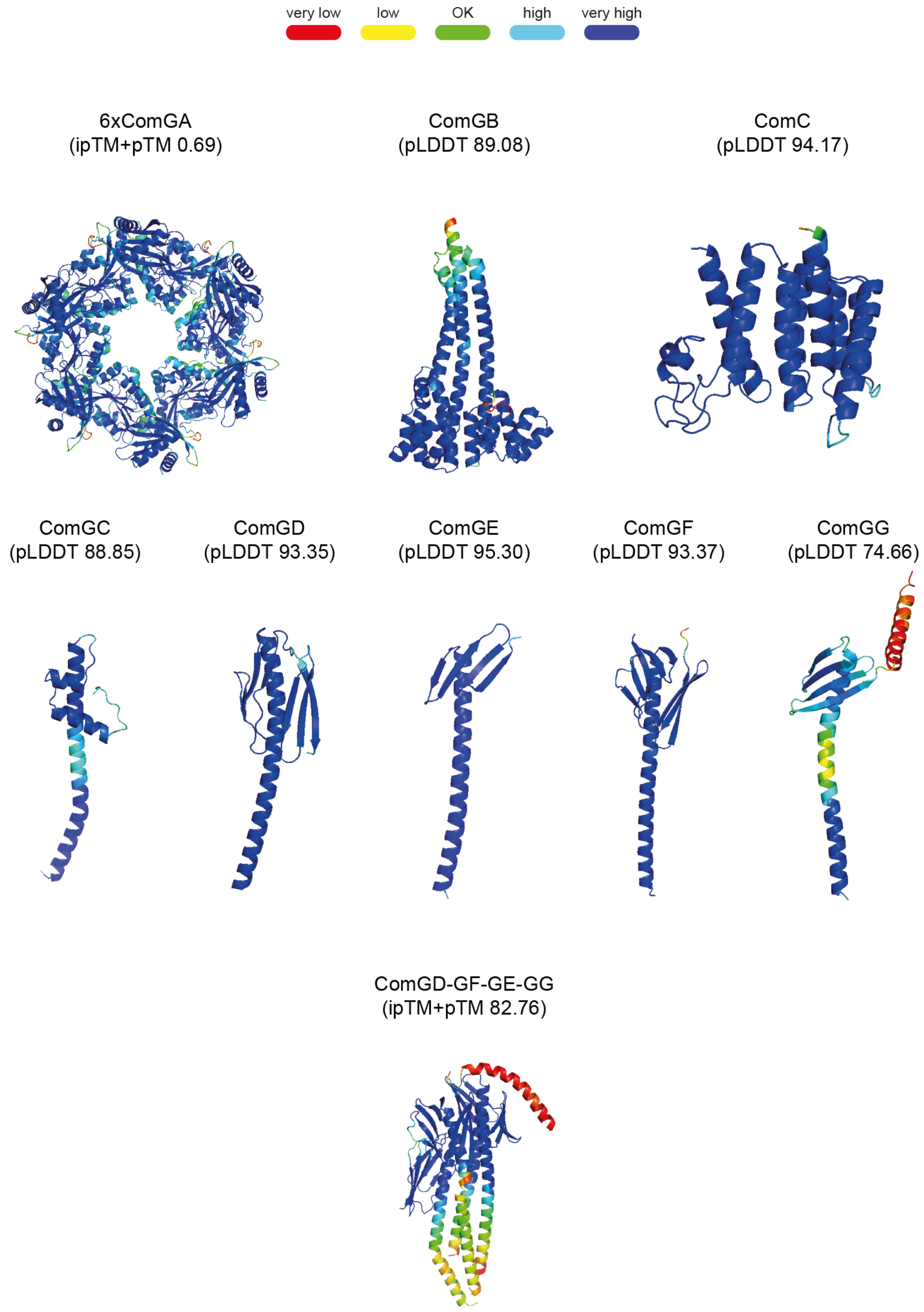

Fig. S5. Accuracy of the AlphaFold models. The per-residue confidence scores given by AlphaFold (3) – pLDDT (monomers) or ipTM+pTM (multimers) ranging from 0 to 100 – are indicated for each model. The structures are colored by confidence measures from dark blue (very high accuracy expected) to red (very low accuracy, which should not be interpreted and may be disordered).

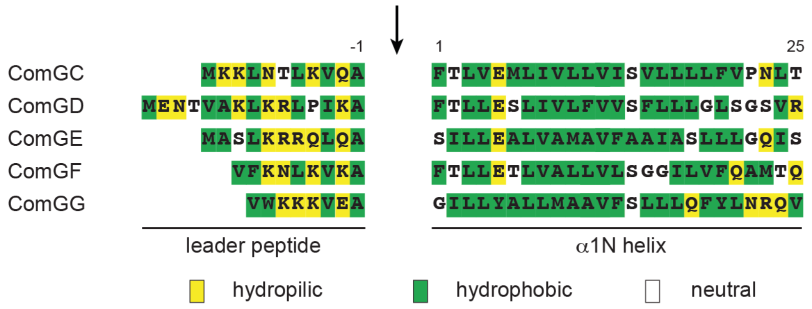

Fig. S6. Sequence alignment of the N-terminal class 3 signal peptides in the five Com pilins in *S. sanguinis* 2908. The 8-15 residues long leader peptides, ending with a conserved Ala, contain a majority of hydrophilic (shaded in yellow) or neutral (no shading) residues. The leader peptides are cleaved (indicated by the vertical arrow) by the PPase ComC. The mature proteins start with a tract of 21 predominantly hydrophobic residues (shaded in green), which form the protruding N-terminal half of an extended α-helix (α1N). α1N is the main assembly interface of pilins within filaments.

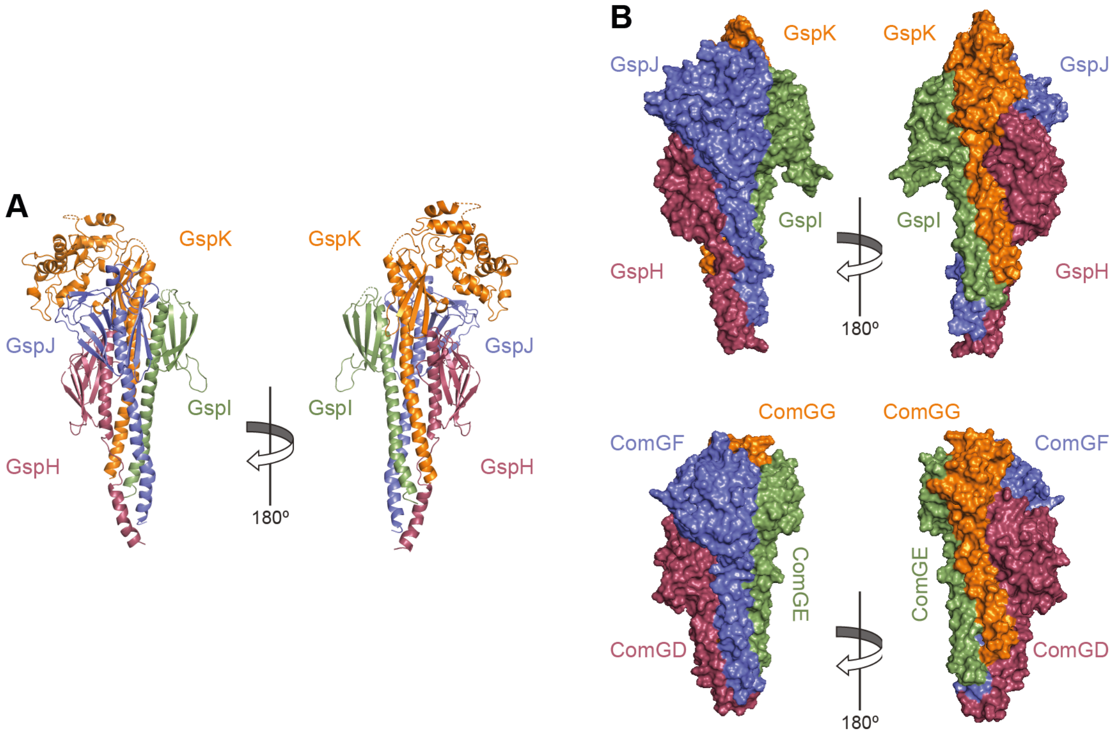

Fig. S7. Structural similarity between tip-located complexes of four minor pilins in Com pili and T2SS. The structures – 180° views – are shown as cartoon or surface. Homologous subunits in the two T4F are highlighted with the same color (we used the same color code as in Fig. 10). (**A**) The GspH-GspJ-GspI-GspK complex from *Pseudomonas aeruginosa* T2SS (4). (**B**) Side-by side comparison of the GspH-J-I-K and ComGD-F-E-G complexes. To better illustrate the structural similarity, we have removed the disordered C-terminus of ComGG and the extra module in GspK.

References

1. Gurung I, Spielman I, Davies MR, Lala R, Gaustad P, Biais N, Pelicic V. 2016. Functional analysis of an unusual type IV pilus in the Gram-positive *Streptococcus sanguinis*. Molecular Microbiology 99:380-392.
2. Okahashi N, Nakata M, Sakurai A, Terao Y, Hoshino T, Yamaguchi M, Isoda R, Sumitomo T, Nakano K, Kawabata S, Ooshima T. 2010. Pili of oral *Streptococcus sanguinis* bind to fibronectin and contribute to cell adhesion. Biochemistry and Biophysics Research Communications 391:1192-1196.
3. Jumper J, Evans R, Pritzel A, Green T, Figurnov M, Ronneberger O, Tunyasuvunakool K, Bates R, Zidek A, Potapenko A, Bridgland A, Meyer C, Kohl SAA, Ballard AJ, Cowie A, Romera-Paredes B, Nikolov S, Jain R, Adler J, Back T, Petersen S, Reiman D, Clancy E, Zielinski M, Steinegger M, Pacholska M, Berghammer T, Bodenstein S, Silver D, Vinyals O, Senior AW, Kavukcuoglu K, Kohli P, Hassabis D. 2021. Highly accurate protein structure prediction with AlphaFold. Nature 596:583-589.
4. Escobar CA, Douzi B, Ball G, Barbat B, Alphonse S, Quinton L, Voulhoux R, Forest KT. 2021. Structural interactions define assembly adapter function of a type II secretion system pseudopilin. Structure 29:1116-1127.
